# Hemoadsorption with CytoSorb shows a decreased observed versus expected 28-day all-cause mortality in ICU patients with septic shock: a propensity-score-weighted retrospective study

**DOI:** 10.1186/s13054-019-2588-1

**Published:** 2019-09-18

**Authors:** Willem Pieter Brouwer, Servet Duran, Martijn Kuijper, Can Ince

**Affiliations:** 1 Department of Gastroenterology and Hepatology, Erasmus MC, University Medical Center Rotterdam, Dr Molewaterplein 15, Building NA-6, 3015 CE Rotterdam, The Netherlands; 2 Department of Internal Medicine, Maasstad Ziekenhuis, Rotterdam, The Netherlands; 3 Department of Intensive Care Medicine, Maasstad Ziekenhuis, Rotterdam, The Netherlands; 4 Science board, Maasstad Ziekenhuis, Rotterdam, The Netherlands; 5 Department of Intensive Care Medicine, Erasmus MC, University Medical Center Rotterdam, Rotterdam, The Netherlands

**Keywords:** Sepsis, Septic shock, Treatment, Hemofiltration, Cytosorb, Cytosorbent, Mortality, Outcome

## Abstract

**Background and aims:**

Innovative treatment modalities have not yet shown a clinical benefit in patients with septic shock. To reduce severe cytokinaemia, CytoSorb as an add-on to continuous renal replacement therapy (CRRT) showed promising results in case reports. However, there are no clinical trials investigating outcomes.

**Methods:**

In this investigator-initiated retrospective study, patients with septic shock were treated with CRRT + CytoSorb (*n* = 67) or CRRT alone (*n* = 49). The primary outcome was the 28-day all-cause mortality rate. Patients were weighted by stabilized inverse probability of treatment weights (sIPTW) to overcome differences in baseline characteristics.

**Results:**

At the start of therapy, CytoSorb-treated patients had higher lactate levels (*p* < 0.001), lower mean arterial pressure (*p* = 0.007) and higher levels of noradrenaline (*p* < 0.001) compared to the CRRT group. For CytoSorb, the mean predicted mortality rate based on a SOFA of 13.8 (*n* = 67) was 75% (95%CI 71–79%), while the actual 28-day mortality rate was 48% (mean difference − 27%, 95%CI − 38 to − 15%, *p* < 0.001). For CRRT, based on a SOFA of 12.8 (*n* = 49), the mean predicted versus observed mortality was 68% versus 51% (mean difference − 16.9% [95%CI − 32.6 to − 1.2%, *p* = 0.035]). By sIPTW analysis, patients treated with CytoSorb had a significantly lower 28-day mortality rate compared to CRRT alone (53% vs. 72%, respectively, *p* = 0.038). Independent predictors of 28-day mortality in the CytoSorb group were the presence of pneumosepsis (adjusted odds ratio [aOR] 5.47, *p* = 0.029), higher levels of lactate at the start of CytoSorb (aOR 1.15, *p* = 0.031) and older age (aOR per 10 years 1.67, *p* = 0.034).

**Conclusions:**

CytoSorb was associated with a decreased observed versus expected 28-day all-cause mortality. By IPTW analysis, intervention with CytoSorb may be associated with a decreased all-cause mortality at 28 days compared to CRRT alone.

**Electronic supplementary material:**

The online version of this article (10.1186/s13054-019-2588-1) contains supplementary material, which is available to authorized users.

## Introduction

Sepsis and septic shock are a major health burden worldwide leading to approximately 5 million deaths annually [1–3]. Although the reported incidence of sepsis varies widely and is notoriously unreliable, it is the leading cause of mortality globally, and its incidence currently is thought to rise due to aging populations, increasing comorbidity and greater recognition due to increasing awareness of this disease over the past decades [4, 5]. Septic shock is thought to arise from a disrupted balance between pro-inflammatory and anti-inflammatory cytokines in response to infection, ultimately leading to cell and organ dysfunction [5]. Recent advances in the field of sepsis treatment had variable success, presumably because sepsis is a very heterogeneous disease entity and therefore resists a one-size-fits-all approach. To date, only advancement in supportive care, such as timely delivery of antibiotics and early fluid resuscitation, has led to a significant improvement in the outcome of sepsis [4]. Other treatment modalities, such as continuous renal replacement therapy (CRRT) have not shown clinical benefit, although it was shown that cytokines such as tumour necrosis factor (TNF) α and interleukin (IL) 1β could be cleared from serum [6–8].

Recently, CytoSorb has been developed and approved in Europe since 2011 for use in patients with severe cytokinaemia [9–11]. CytoSorb is a filter which can be used in addition to continuous renal replacement therapy (CRRT), and other devices such as hemodialysis, heart-lung machines and extracorporeal membrane oxygenation. It is a non-pyrogetic, sterile single-use filter for the removal of endotoxins and cytokines [10]. Since it is able to reduce circulating cytokines such as IL-1β, TNF-α, IL-6 and IL-10 by more than 90%, CytoSorb is thought to have considerable impact on a derailed host response causing shock [10, 12]. This treatment modality has shown promising results in animal studies [13, 14] and case reports [15–18]. Nevertheless, a recent randomized trial in patients with septic shock and acute lung injury (ALI) or acute respiratory distress syndrome (ARDS) assessed, but was not powered for mortality, and found no difference in clinical outcome [11, 19]. It is therefore unknown whether CytoSorb leads to a survival benefit.

Hence, the aim of the current study is to investigate whether the application of CytoSorb in addition to CRRT leads to a reduction in 28-day mortality compared to CRRT alone in patients with septic shock in the ICU, by using the inversed probability of treatment weights method.

## Patients and methods

### Patients

In this retrospective investigator-initiated study, patients admitted to the ICU of the Maasstad Hospital with septic shock [5] treated with CRRT with or without CytoSorb from Jan 01, 2014 - April 01, 2017, were initially eligible for inclusion. CytoSorb was initiated at the discretion of the treating intensive care physician. Indications for CytoSorb therapy were age 18–80 years and having a septic shock (see definitions below). Patients were treated per protocol, agreed upon by the staff of intensive care physicians. All patients in this study were treated with CRRT. Patients were excluded from the analysis in case the primary diagnosis was not septic shock (out of hospital cardiac arrest, rhabdomyolysis, intoxications, metabolic disturbances, kidney or heart failure with type 1 respiratory insufficiency requiring CRRT, or presence of active malignancy). Moreover, CytoSorb or CRRT was discontinued in case shock or renal function was improved. For the current study, to test the application of CytoSorb to CRRT in a clinical practice setting, there were no constraints to the timing of admission to the ICU, the severity of septic shock at the start of therapy and the eventual duration of therapy. Patients were treated per protocol as part of standard of care, i.e. no interventions were applied for the purpose of this study, and data was collected retrospectively. Patients who initiated on CytoSorb subsequent to CRRT were evaluated in the CytoSorb cohort. CytoSorb was used according to the manufacturer’s protocol. It was placed in a blood-pump circuit with an optimal ultrafiltration rate of 250–400 mL/min. The CytoSorb filter was changed after 24 h of use.

### Definitions

Septic shock was defined as a life-threatening organ dysfunction caused by a dysregulated host response to infection, identified by persisting hypotension requiring vasopressive medication to maintain mean arterial pressure (MAP) ≥ 65 mmHg and having a serum lactate level > 2 mmol/L despite adequate volume resuscitation [5]. Shock reversal was defined as a serum lactate level ≤ 2 mmol/L and discontinuation of vasopressive medication [20].

### Endpoints

The primary endpoint was the 28-day all-cause mortality compared for CytoSorb versus CRRT alone. Secondary endpoints included the comparison between the observed 28-day mortality rate in the CytoSorb treatment group versus the predicted mortality according to the SOFA score [21, 22], and variables that predict mortality in the CytoSorb group. All-cause mortality was measured from ICU admission until 28 days after admission (irrespective of ICU, in-hospital or out of hospital mortality).

### Statistical analysis

For the first part, the predicted probability according to the SOFA score at the start of therapy [21, 22] was calculated for each individual and compared with the observed mortality rate in the CytoSorb group using a paired *T*-test.

For the second part, all evaluations were carried out using the inverse probability of treatment weights (IPTW), including a stabilizing method to avoid bias from extreme weights [23, 24]. Stabilized IPTW (sIPTW) is applied to overcome differences in baseline patient characteristics, to mimic a randomized controlled trial. Weights are based on the propensity score to create a synthetic sample in which the baseline variables are independent of the treatment assignment. First, all variables in the dataset were tested for their association with either CytoSorb or CRRT treatment. From this analysis, factors associated with the treatment in univariate analysis were selected to construct a multivariable model to estimate the probability of being treated with CRRT or CytoSorb. Second, the probability of being treated with CRRT or CytoSorb was estimated using the following baseline factors in a logistic regression analysis: age, SOFA score at the start of therapy, lactate level at the start of therapy, dosage of vasopressive medication (in μg/kg/min) at the start of therapy, known comorbidity, surgery just prior to or during ICU admission and origin of sepsis. These factors are also associated with the primary outcome [5, 21, 25]. Third, patients were weighted by the inverse of this propensity, which was stabilized prior to the analyses using the estimated marginal means of the calculated propensity [23]. The absolute standardized difference for variables between the two treatment groups were calculated using Cohen’s D and graphically inspected (Additional file 1: Figure S1). The association between CytoSorb or CRRT with clinical outcome at 28 days was then estimated by chi-square analysis. Fourth, to account for variables still showing imbalance after adjustment by sIPTW, multivariable logistic regression was applied using both the stabilized weights and adjusting for unbalanced variables [24]. Factors associated with 28-day mortality in CytoSorb-treated patients were analysed as well. For this, factors with a *p* value < 0.1 in univariate analysis were considered for multivariable regression analyses. First, a full multivariable model was constructed where all possible important variables were forced into the model. Second, using the same variables from the full model, the final model was constructed with the backstep likelihood ratio method. SPSS version 22.0 (SPSS Inc., Chicago, IL, USA) was used to perform statistical analyses. All statistical tests were two-sided and evaluated at the 0.05 level of significance.

## Results

### Patient characteristics

**Fig. 1:**
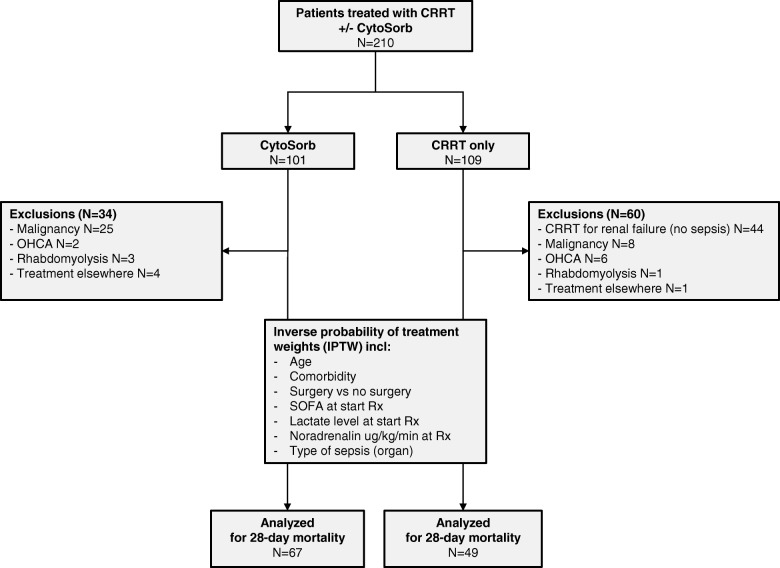
Patient and analysis flowchart

In total, 210 patients were treated, of which 101 with CytoSorb and 109 with CRRT only. Of the patients treated with CytoSorb, 67 were treated because of septic shock, versus 49 for CRRT. Figure 1 shows the patient selection and reasons for exclusion. The patient characteristics can be found in Table 1. It was observed that patients treated with CytoSorb had worse hemodynamic characteristics when compared to CRRT alone. CytoSorb-treated patients had higher lactate levels both at admission (*p* = 0.027) and at the start of therapy (*p* < 0.001), were administered higher levels of noradrenaline (*p* < 0.001) and had lower mean arterial pressure (*p* = 0.007). Patients were treated with CRRT for a mean duration of 4.96 (SE 0.63) days in the CRRT group and 4.97 (SE 0.55) days in the CytoSorb group (*p* = 0.990). In the CytoSorb group, patients were treated with CytoSorb added to CRRT for a mean duration of 2.34 (SE 0.16) days and with CRRT only for a mean 2.66 (SE 0.52) days. The mean duration from ICU admission to the start of treatment was 2.1 (SE 0.36) versus 1.66 (SE 0.38) days (*p* = 0.416) for CRRT versus CytoSorb, respectively. In total, 88% (*n* = 59) patients commenced CytoSorb directly together with CRRT because of septic shock (i.e. no delay between CRRT and CytoSorb), 4 patients had a delay in the start of CytoSorb after CRRT of 1 day and another 4 had a delay of > 1 day. Moreover, the mean duration from hospital admission to the start of treatment did not differ between the groups (4.7 versus 3.8 days, *p* = 0.306). After adjustment with sIPTW, the CRRT and CytoSorb groups were largely comparable (Table 1 and Additional file 1: Figure S1).

**Table 1:** Patient characteristics (at ICU admission and at the start of CytoSorb or CRRT)

Characteristics	CytoSorb (*N* = 67)	CRRT only (*n* = 48)	Unadjusted *p* value	sIPTW-adjusted *p* value
Demography				
Age, years (Mean, SD)	61.1 (14.7)	68.7 (9.6)	0.001	0.126
Male, *n* (%)	37 (55%)	30 (61%)	0.522	0.021
Comorbidity, *n* (%)				
Any comorbidity	43 (64%)	42 (88%)	0.002	0.601
Diabetes mellitus type 2	14 (21%)	20 (41%)	0.024	0.778
Hypertension	23 (34%)	26 (53%)	0.046	0.409
Coronary heart disease	9 (13%)	8 (16%)	0.667	0.064
Heart failure (systolic/diastolic)	4 (6%)	10 (20%)	0.030	0.501
Prior chronic kidney disease	8 (12%)	18 (37%)	0.003	0.002
Peripheral artery disease	10 (15%)	8 (16%)	0.839	0.021
Cerebrovascular accident	6 (9%)	3 (6%)	0.577	0.369
COPD	8 (12%)	10 (20%)	0.233	0.002
Primary diagnosis, *n* (%)				
Abdominal sepsis	31 (46%)	12 (25%)	0.014	0.870
Pneumosepsis	14 (21%)	21 (43%)	0.014	0.275
Urosepsis	2 (3%)	6 (12%)	0.078	0.217
Cutaneous/arthritis	9 (13%)	3 (6%)	0.182	0.064
Vascular sepsis	5 (8%)	0	0.024	0.026
Cerebral sepsis	0	1 (2%)	0.322	0.475
Sepsis (unknown cause)	6 (9%)	6 (12%)	0.569	0.755
Admission				
Surgical (otherwise medical)	27 (40%)	6 (12%)	< 0.001	0.994
Days on ICU	9 (2–19)	9 (3–13)	0.783	0.463
Hemodynamics (Mean, SD)				
Lactate at admission	6.4 (5.1)	4.4 (4.4)	0.027	0.421
Lactate at the start of therapy	6.9 (5.6)	2.9 (3.1)	< 0.001	0.544
Noradrenaline (μg/kg/min) adm.	0.48 (0.55)	0.29 (0.40)	0.052	0.073
Noradrenaline (μg/kg/min) Rx	0.96 (0.73)	0.28 (0.36)	< 0.001	0.769
Total duration noradrenaline (days)	3 (1–5)	3 (1–5)	0.979	0.222
Duration noradrenaline from Rx (days)	2 (1–3)	1 (0–2)	0.694	0.989
MAP at admission	73 (19)	74 (20)	0.748	0.418
MAP at the start of therapy	69 (15)	77 (18)	0.007	0.019
Prognostic scores				
SOFA ICU admission	11.7 (3.3)	11.8 (3.5)	0.907	0.854
SOFA at the start of treatment	13.8 (2.8)	12.8 (3.2)	0.067	0.239

### CytoSorb treatment: observed versus predicted 28-day mortality

**Fig. 2:**
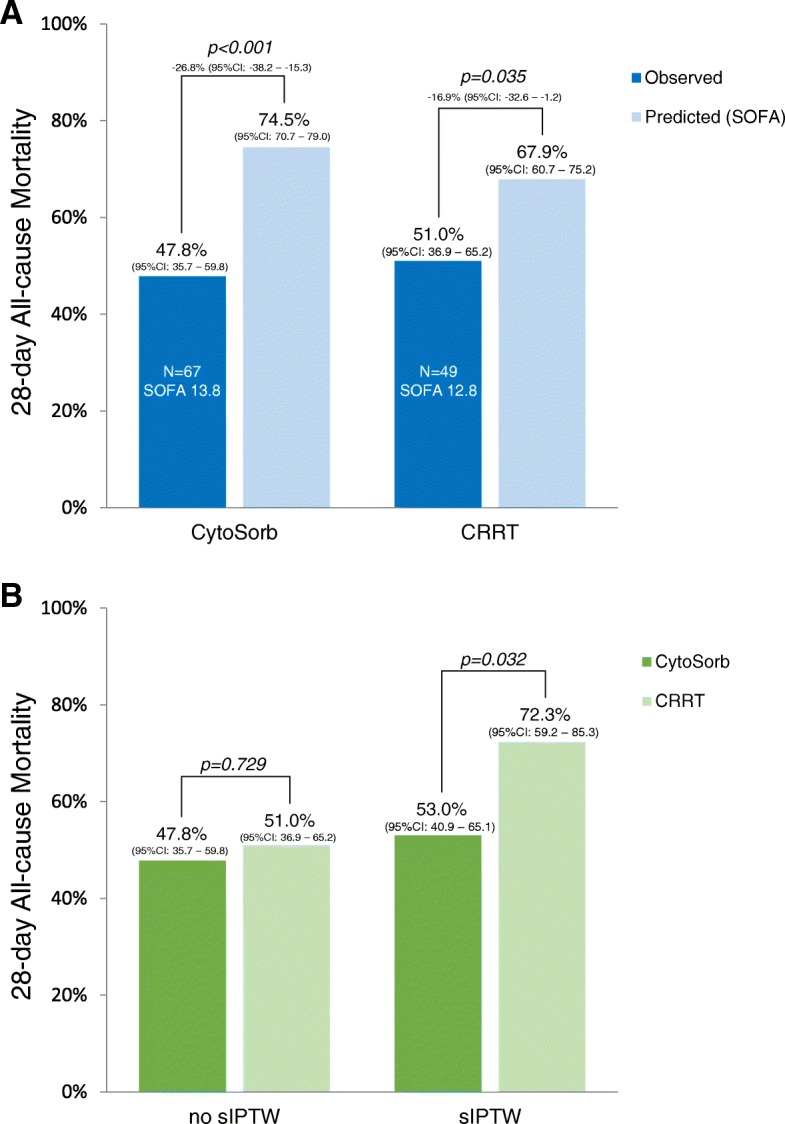
**a** Observed versus predicted mortality rate according to the SOFA score for CytoSorb- and CRRT-treated patients. **b** CytoSorb is associated with a reduced 28-day mortality in sIPTW analysis

When no correction for baseline variables was applied, it was shown that the 28-day all-cause mortality rate was similar for CytoSorb versus CRRT (47.8% versus 51.0%, *p* = 0.729, Fig. 2). For the CytoSorb group, the mean SOFA score at the start of therapy was 13.8 (SE 2.8), and the delta SOFA score (admission to treatment) was 2.1 (SE 0.41). On the basis of the SOFA score, the mean predicted mortality rate was 74.5% (95%CI 70.7–79.0%) [21], while the actual 28-day mortality rate was 47.8% (95%CI 35.7–59.8%), corresponding to a mean difference of − 26.8% (95%CI − 38.2 to − 15.3%, *p* < 0.001). For the CRRT only group, the mean SOFA score was 12.8 (SE 3.2), corresponding to a mean predicted mortality rate of 67.9% (95%CI 60.7–75.2%), while the observed mortality rate was 51.0% (95%CI 36.9–65.2%) with a mean difference − 16.9% (95%CI − 32.6 to − 1.2%, *p* = 0.035).

### CytoSorb is associated with a reduced 28-day mortality: sIPTW analysis

By sIPTW chi-square analysis, the 28-day mortality significantly differed for CytoSorb versus CRRT: 53.0% versus 72.3%, respectively (*p* = 0.038, Fig. 2). By sIPTW multivariable analysis additionally adjusted for MAP and chronic kidney disease (CKD), CytoSorb treatment was also significantly associated with an improved 28-day outcome (OR 0.37, 95% confidence interval [CI] 0.15–0.92, *p* = 0.032). In this analysis, CKD was not associated with 28-day mortality (OR 1.93, 95%CI 0.69–5.44, *p* = 0.214), and a higher MAP was associated with a lower chance of mortality (OR 0.95, 95%CI 0.92–0.98, *p* = 0.003).

### Factors associated with 28-day mortality for CytoSorb therapy

Factors at the start of therapy significantly associated with mortality at 28 days for CytoSorb-treated patients were the SOFA score (OR 1.28, 95%CI 1.05–1.56, *p* = 0.014), lactate levels (OR 1.16, 95%CI 1.03–1.30, *p* = 0.014), noradrenaline levels (OR 2.60, 95%CI 1.15–5.87, *p* = 0.021), and older age (OR 1.67 per 10 years older, *p* = 0.002, Table 2). For patients with mortality versus those who survived, initiation of CytoSorb was 1.13 (SE 0.35) versus 2.14 (SE 0.65) days, respectively (*p* = 0.18). To investigate whether CytoSorb treatment may have been initiated earlier in patients with worse septic shock, interactions between therapy timing and lactate levels or SOFA score at the start of therapy were applied, which were found non-significant (*p* = 0.538 and *p* = 0.930, respectively). By multivariable analysis, independent predictors of 28-day mortality in the CytoSorb group were older age (OR per 10 years older 1.67, 95%CI 1.00–2.70, *p* = 0.034), higher levels of lactate at the start of therapy (OR 1.15, 95%CI 1.01–1.30, *p* = 0.031) and pneumosepsis (OR 5.47, 95%CI 1.19–25.19, *p* = 0.029). Other factors were not independently associated with mortality (Table 2).

**Table 2:** Regression analysis for mortality at 28 days for CytoSorb-treated patients

Variables	Univariable		Full multivariable model (forced)		Final multivariable model (bstep LR)	
	OR (95% CI)	*p*	OR (95% CI)	*p*	OR (95% CI)	*p*
*Age per 10 years older*	*1.99 (1.3–3.1)*	*0.002*	*1.64 (1.0–2.7)*	*0.050*	*1.67 (1.0–2.7)*	*0.034*
Female gender	1.50 (0.6–4.0)	0.412	–		–	
Body mass index	0.96 (0.9–1.0)	0.260	–		–	
SOFA admission	1.20 (1.0–1.4)	0.041	–		–	
*SOFA at the start of treatment*	*1.28 (1.1–1.6)*	*0.014*	1.10 (0.8–1.4)	0.505	–	
MAP admission	0.99 (0.9–1.0)	0.460	–		–	
*MAP at the start of treatment*	*0.96 (0.9–1.0)*	*0.051*	0.99 (1.0–1.03)	0.739	–	
Lactate admission	1.07 (0.9–1.2)	0.180	–		–	
*Lactate at the start of treatment*	*1.16 (1.0–1.3)*	*0.014*	*1.13 (1.0–1.3)*	*0.108*	*1.15 (1.0–1.3)*	*0.031*
Noradrenaline admission	2.25 (0.8–6.0)	0.106	–		–	
*Noradrenaline treatment*	*2.60 (1.2–5.9)*	*0.021*	0.98 (0.3–2.8)	0.973	–	
Noradrenaline duration	1.02 (0.9–1.2)	0.731	–		–	
Admission ICU to treatment	0.88 (0.7–1.1)	0.216	–		–	
Admission hospital to treatment	0.98 (0.9–1.1)	0.675	–		–	
*Surgery*	*0.37 (0.1–1.0)*	*0.055*	0.43 (0.1–1.6)	0.206	–	
Type of sepsis:	–		–		–	
*Pneumosepsis*	*3.52 (1.0–12.7)*	*0.054*	2.83 (0.5–16.9)	0.254	*5.47 (1.2–25.2)*	*0.029*
Abdominal sepsis	0.51 (0.2–1.3)	0.171	–		–	
Cutaneous sepsis	0.27 (0.1–1.4)	0.117	–		–	
Vascular sepsis	4.86 (0.5–46.0)	0.168	–		–	
Sepsis unknown cause	2.36 (0.4–13.8)	0.343	–		–	
History of:	–		–		–	
Diabetes mellitus	1.61 (0.5–5.3)	0.432	–		–	
Hypertension	1.31 (0.5–3.6)	0.601	–		–	
COPD	1.97 (0.4–9.0)	0.380				
Coronary artery disease	0.86 (0.2–3.5)	0.831	–		–	
Heart failure	NA	0.999				
Chronic kidney disease	1.98 (0.4–9.0)	0.380	–		–	
Hemodialysis/peritoneal	1.10 (0.1–18.3)	0.949	–		–	
CVA	1.10 (0.2–5.9)	0.908	–		–	
Peripheral artery disease	2.99 (0.7–12.7)	0.139	–		–	

*MAP* mean arterial pressure, *ICU* intensive care unit, *COPD* chronic obstructive pulmonary disease, *CVA* cerebrovascular accident

Statistically significant data are italicized

## Discussion

In this investigator-initiated retrospective study, we have shown for the first time that CytoSorb therapy may improve the 28-day mortality for patients with septic shock, compared to CRRT. To our knowledge, this represents the largest cohort of septic shock patients treated with CytoSorb therapy in which mortality was assessed as a primary outcome. The observed mortality rate for CytoSorb therapy was significantly below the predicted risk of death according to the SOFA score [21, 22]. For CytoSorb, factors associated with a higher chance of 28-day mortality were older age, higher lactate levels at the start of therapy and pneumosepsis.

In the current study, it was investigated whether CytoSorb improves survival when compared to CRRT alone in an IPTW analysis. Per protocol, patients were only treated with CytoSorb in case of septic shock, at the discretion of the treating intensive care physician. As a result, it was found that at the start of therapy, patients treated with CytoSorb had a worse septic shock than those treated with CRRT alone as shown by higher lactate and noradrenaline levels, and lower mean arterial blood pressure. Despite that CytoSorb-treated patients had a worse shock, in an unbalanced analysis, the mortality rate was comparable to patients treated with CRRT alone. However, it could be argued that patients treated with CRRT alone were older with more comorbidities and more often having non-surgical sepsis. Therefore, observed versus predicted mortality rates were analysed within treatment groups. This analysis showed that the mortality rates in this study were lower than that predicted by the SOFA score at the start of therapy [21]. The results of our study are in line with a previous prospective study in 20 patients with septic shock treated with CytoSorb, where a 28-day mortality rate of 45% was reported as well [20].

It is important to investigate factors which are associated with survival for CytoSorb treatment. For the current cohort, next to older age, we found that higher lactate levels at baseline of CytoSorb therapy were associated with a worse outcome. Indeed, these factors are components of the SOFA score itself. Apparently, particularly the measures of the hemodynamic components of the SOFA score showed the strongest association with outcome. Hence, it seems reasonable to assume that CytoSorb therapy should be initiated as early as possible in the disease course. Nonetheless, 8 (12%) patients received CytoSorb at least 1 day after CRRT was already started. We performed a sensitivity analysis in which we excluded these patients and found no deviations from the main results.

In our cohort, it was found that CytoSorb therapy did not seem to provide with a survival benefit for patients with pneumosepsis. This may in part be due to combined difficulties in adequate ventilation and/or presence of ALI or ARDS in these severely ill patients. These findings are underlined by a recent randomized trial which assessed IL-6 levels as a primary outcome in patients with ALI and ARDS and found no survival benefit in these patients as a secondary (but not powered for) outcome measure [11].

Since the current study is a retrospective data analysis, there is inherent bias to take into account. Importantly, by applying IPTW, confounding by indication was as much as possible eradicated [24]. Still, chance of residual confounding remains, and some variables were imbalanced at the start of therapy. Only MAP was associated with the primary endpoint, which was accounted for by multivariable analysis. Moreover, the precise amount of fluid balances and ultrafiltration rates were not available for both treatment groups, which was complicated because this would need a dynamic statistical analysis. Indeed, fluid balances may be important measures as several studies show an association with more positive fluid balances and mortality [26, 27], but there are also controversial findings with studies showing an association between higher fluid balance and improved survival [28, 29], and even no differences in early goal-directed therapy [30–32]. It should be underlined that it is uncertain whether the association between a positive fluid balance and mortality is a true dose-response causal relationship. Even though the omission of data on the fluid balance may be a limitation of the study, both groups received exactly the same standard of care fluid resuscitation protocols since this is a mono-centre study. On this basis, both groups are not expected to differ accordingly.

Albeit patients had a severe refractory septic shock and were treated with CytoSorb as a last -resort, one may argue that this treatment modality could have potential detrimental effects. One of these effects may be that CytoSorb could filter out antibiotics leaving patients exposed to levels below the therapeutic range [33]. In the current study, we did not have antibiotic levels available. Nonetheless, there were no observations or indications of excessive need for antibiotics or persistence of infections in the CytoSorb group. Moreover, septic shock originates from a severe host-response derailment and endotoxinaemia and as such these patients may benefit more from CytoSorb therapy than antibiotics alone, which is underlined by the current data. Our data also shows that CytoSorb leads to a better outcome in patients with less severe lactataemia. Given possible antibiotic filtration, caution is warranted since the positive effect may be tipped towards a more negative effect if CytoSorb therapy is initiated too early. Lastly, a recent pilot study showed positive effects on lactate and procalcitonin when CytoSorb was used as a stand-alone therapy [34]. We did not have procalcitonin or interleukin levels available in our study. Future randomized trials comparing CytoSorb to CRRT should further elucidate the effect on interleukin, procalcitonin and antibiotic levels, and what the timing and duration of CytoSorb therapy should be.

## Conclusion

We have shown, to our knowledge, in the largest cohort of septic shock patients to date, that CytoSorb treatment may lead to an improved 28-day survival compared to CRRT alone, both on basis of observed versus predicted mortality rates as well as by IPTW. The current data should be further corroborated by randomized clinical trials.

## Additional file

Additional file 1: **Figure S1.** Absolute standardized differences between CRRT and CytoSorb for variables before and after weighting. (PDF 403 kb)

## Data Availability

The dataset used and analysed during the current study are available from the corresponding author on reasonable request.

## Abbreviations

ALI: Acute lung injury
ARDS: Acute respiratory distress syndrome
CKD: Chronic kidney disease
CRRT: Continuous renal replacement therapy
ECMO: Extracorporeal membrane oxygenation
IL: Interleukin
MAP: Mean arterial pressure
sIPTW: Stabilized inverse probability of treatment weights
ICU: Intensive care unit
OR: Odds ratio
LR: Likelihood ratio
SD: Standard deviation
SE: Standard error
TNF: Tumour necrosis factor
