# Supplementary material for: Hemoadsorption with CytoSorb shows a decreased observed versus expected 28-day all-cause mortality in ICU patients with septic shock: a propensity-score-weighted retrospective study
Source: Crit Care. 2019 Sep 18;23:317. doi: 10.1186/s13054-019-2588-1 (PMC6749645; doi:10.1186/s13054-019-2588-1)
Supplement: Supplementary file 1 — Figure S1. Absolute standardized differences between CRRT and CytoSorb for variables before and after weighting. (PDF 403 kb) [file 13054_2019_2588_MOESM1_ESM.pdf]

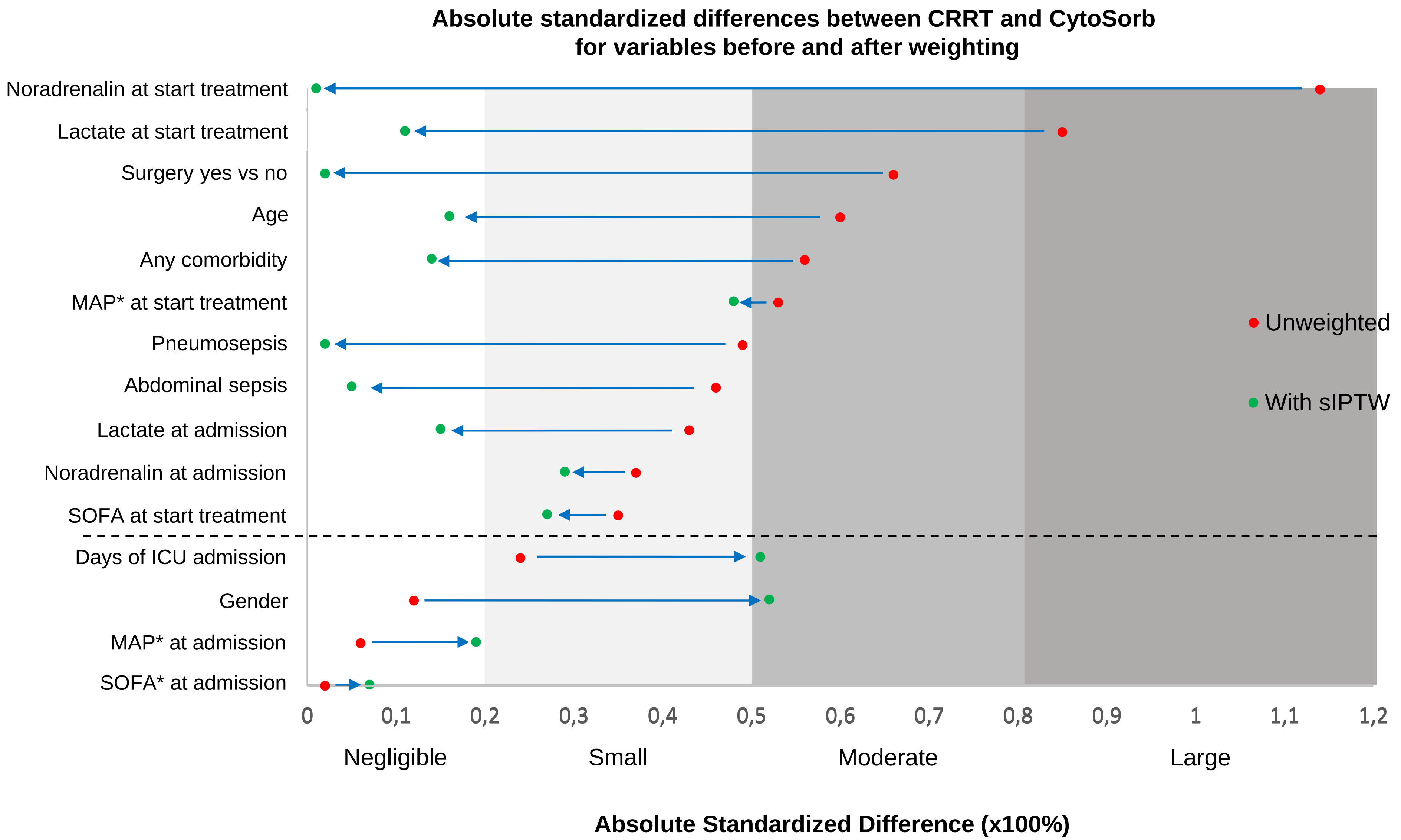

Red rounds represent the absolute standardized difference for the variables of interest without applying weights. Green rounds represent the absolute standardized difference after sIPTW. Blue arrows show the direction of improvement (right to left) or detoriation (left to right). Moreover, the white – grey areas represent the significance of the difference observed according to Cohen et al. (1977). *MAP, mean arterial pressure.

sIPTW characteristics:

Median weight CRRT: 0.424, interquartile range 0.385 – 0.615
Median weight CytoSorb: 0.717, interquartile range 0.669 – 0.994
